# SLEEP DISORDERS AND MUSCLE STRENGTH IN OLDER MEXICAN AMERICANS: SEX DIFFERENCES

**DOI:** 10.1093/geroni/igad104.3592

**Published:** 2023-12-21

**Authors:** Jamil Okada, Soham Al Snih

**Affiliations:** University of Texas Medical Branch, Galveston, Texas, United States; University of Texas Medical Branch, Galveston, Texas, United States

## Abstract

We used data from the Hispanic Established Population for the Epidemiological Study for the Elderly to examine sex differences in the relationship between sleep duration and handgrip strength (HGS) among Mexican Americans aged ≥ 77 years with normal or high HGS (≥ 17 in women and ≥ 27 in men) at baseline over a 9-year period (2007/08 to 2012/13; N=1542). Measures included socio-demographics, sleep duration, HGS (Kg), body mass index, medical conditions, depressive symptoms, physical function, cognitive function, disability, and pain. We categorized sleep duration as ≤ 7, 8, 9, and ≥ 10 hours/night and used linear mixed models to estimate the changes in HGS as a function of sleep duration by sex, adjusting for covariates. Both men and women in the highest sleep duration group (≥10 hours/night) had on average the greatest decline in HGS (1.54 kg and 0.95 kg, respectively) compared to those who slept 8 hours/night, after controlling for all covariates over the follow-up period. Older age, time (years), depressive symptoms, one or more falls in the last year, and low physical function were associated with greater decline in HGS among women. Older age and time (years) were associated with greater decline in HGS among men. In conclusion, longer sleep duration was associated with greater loss of HGS over time in older Mexican Americans. Sleep health promotion and sleep behavioral interventions may reduce the risk of decline in HGS, which has been associated with disability, institutionalization, and mortality in this population.

